# The impacts of Covid-19 lockdowns on professional and personal lives of freelance creative collaborative musicians

**DOI:** 10.1177/1321103X231186952

**Published:** 2023-07-21

**Authors:** Katie Zhukov, Margaret S Barrett, Andrea Creech

**Affiliations:** Monash University, Australia; Monash University, Australia; McGill University, Canada

**Keywords:** lockdowns, musicians, pandemic, performing arts, resilience

## Abstract

The global pandemic has severely disrupted the performing arts sector, with research documenting economic, professional, and health impacts on musicians. The psychological effects of lockdowns have been recognized, but little is known regarding their impact on freelance creative collaborative artists. This qualitative case study uses a resilience lens to report the perspectives of freelance creative collaborative musicians from the city of Melbourne, the Australian city which experienced the greatest period of lockdown in the country. Three main themes were identified: professional impacts (loss of work, loss of artistic identity, professional coping strategies), personal impacts (lockdown stressors, personal coping strategies, relationships), and future professional outlook (developing new professional skills and directions, positive and negative future outlooks). The findings demonstrate these musicians’ resilience in spite of difficult circumstances, resulting in positive adaptations and personal growth.

## Introduction

Since the start of the worldwide Covid-19 pandemic in early 2020, performing artists have been significantly affected by public policies of social distancing and community lockdowns leading to cancellations of concerts, live performances, theater, dance, and musicals. This in turn has produced a body of research publications dating from 2021, investigating the impact of the pandemic on the professional and personal lives of performers.

Even before the pandemic, performing arts sector careers were precarious. A longitudinal case study of eight freelance brass musicians in the United Kingdom showed that a portfolio career in the 21st century involves “concurrent multiple job roles” and, in addition to musical expertise, requires “other softer skills such as the ability to network both formally and informally, be entrepreneurial as well as use forms of social media as a method of modern-day promotion” (Gee & Yeow, 2021, p. 351). A recent meta-analysis of published surveys of creative cultural workers in the United Kingdom revealed critical issues such as the perceived impact of the pandemic on immediate changes in revenue, short-term economic viability, and financial support needed (Comunian & England, 2020). The authors argued that “Covid-19 is an emergency that exacerbates the precarious structural conditions of the sector” and called for “long-term structural changes in the sector and its working conditions” (p. 122). Interviews with early-career arts workers in the United Kingdom revealed that “pandemic disruptions accentuated the precarity of their professional lives” (Shaughnessy et al., 2022, p. 4). Similar issues are being reported from the United States, with research into community radio highlighting the impacts of the pandemic on practitioners:

the pandemic and the entrenched inequalities it has revealed has demonstrated that our experiences of the pandemic were and are hugely diverse, determined by financial and physical security or precarity, the nature of our work, whether we had children or others to be cared for. (Moylan, 2023, p. 13)

The impact of the pandemic on the Australian arts industry was documented in the Australia Institute’s report *Creativity in Crisis*, describing losses of 80,000 jobs and $24 billion in income (Pennington & Eltham, 2021). The federal government provided some financial support to large national and state opera companies, symphony orchestras, ballet companies, theater companies, and medium-sized arts organizations through arts packages. The government policy JobKeeper subsidized the salaries of workers who had been working in organizations for longer than 12 months. Unfortunately, this policy excluded many in the arts industry who work on casual and short-term contracts. The JobKeeper support program was wound down in March 2021 despite continuing lockdowns in major cities such as Sydney and Melbourne owing to the Delta wave of the Covid-19 pandemic. *Creativity in Crisis* concluded that the “government approach to the arts has been insufficient in guaranteeing its long-term stability and vitality” (Pennington & Eltham, 2021, p. 5). The authors advocated for a complete reboot of arts funding in Australia, including “large fiscal investments to help rebuild skills, jobs and incomes in the cultural sector, long-term funding for arts organisations and artists, wage subsidies, intervention in cultural regulations, and a holistic plan for culture across the nation” (p. 6). In Western Australia, a report into challenges faced by the performing arts sector during the first half of 2020 identified loss of income and work opportunities, increased psychological distress, and negative impact on well-being (Rusak et al., 2021). Similarly, a survey of 40 creative arts workers in Victoria between August and October 2020 found “creative arts as a devalued and often ignored sector and industry in Australia” and that “the sector is misunderstood by decision-makers” (Flore et al., 2023, p. 203).

Musicians tend to rely on performances as the main source of income, making lockdowns particularly disruptive. A survey of 385 performing musicians in the United Kingdom during the first wave of the pandemic in April to June 2020 examined the impact of Covid-19 on their work, health, and well-being (Spiro et al., 2021). The results showed substantial reduction in work and income, resulting in financial hardship and increased anxiety and loneliness. Those who were more physically active prior to lockdown exhibited “higher wellbeing and social connectedness scores, as well as lower loneliness scores” (p. 1). Thematic analyses of open text identified five themes: “loss or uncertain work and income; constraints of lockdown working; loss and vulnerability; detrimental effects on health and wellbeing; and professional and personal opportunities” (p. 1). Similar themes were identified in interviews with 12 mid-career and 12 veteran freelance orchestral musicians in the United Kingdom (Cohen & Ginsborg, 2021). Five over-arching themes were reported: loss of career, anxiety, maintaining identity as a musician, strategies of coping, and opportunities and positives. Differences between the two groups emerged, with mid-career musicians appearing to be “less motivated to practice, less engaged in collaborative music making, getting less pleasure from the music making that they were doing and less emotionally robust than their seasoned colleagues” (p. 11). However, a follow-up 1 year later showed that “mid-career participants remained committed to their performing careers” while the older musicians had virtually retired (Cohen & Ginsborg, 2022, p. 12). The Melbourne Digital Concert Hall in Australia was one initiative that allowed artists to continue performing on a digital platform (Vincent, 2023). While the program had “partially addressed three negative impacts caused by the pandemic—the losses of work, identity, and community—it was unable to counteract a fourth negative impact—the loss of audience—due to its digital format” (p. 1).

Widespread lockdowns in the eastern states of Australia significantly affected the freedom of movement, with people infected with Covid-19 required to isolate for 14 days in their own home or a medi-hotel. Melbourne has achieved the title of the most-locked-down city in Australia by amassing 262 days of isolation across multiple lockdowns during 2020 to 2021. The Victorian government policies included nightly curfews, 5-km travel limits for basic necessities and medical assistance, 1-hr daily exercise limits, and restrictions on social gatherings. Creative arts workers in Victoria reported that “physical restrictions isolated participants from communities that fostered their social, emotional and mental health” (Flore et al., 2023, p. 206).

The professional needs of small-to-medium freelance creative collaborative groups (Barrett et al., 2021) that generate their own performance opportunities as means of presenting new and innovative work were not recognized and that sector of the creative industry received no support from the Australian federal government. Therefore, the impacts of recurring lockdowns on creative collaborative musicians’ professional and personal lives merit a specific exploration. To remedy this lack of understanding, the current study investigated the following research questions:

1. What has been the impact of recurring lockdowns on the professional lives of freelance creative collaborative musicians?
2. What has been the impact of recurring lockdowns on the personal lives of freelance creative collaborative musicians?
3. How has the impact of recurring lockdowns affected their future professional outlook?

## Theoretical framework

Dealing with major disasters (fires, floods, pandemics) requires resilience. The theory of resilience has been a focus of research since the early 1990s, and its conceptualization has changed over time. For example, Fletcher and Sarkar (2013) in their critique of definitions, concepts, and theory of psychological resilience state that “most definitions are based around two core concepts: adversity and positive adaptation” and that resilience can be viewed as a trait or a process (p. 12). They suggest that “resilience is conceptualized as the interactive influence of psychological characteristics within the context of the stress process” (p. 12). Similarly, Mahdiani and Ungar (2021) explain that “the term is typically used to describe fortitude under stress that results in socially expected and desirable outcomes,” but “use of the term resilience has also been misapplied to situations where the situation might more accurately have been described as producing vulnerability” (p. 151). A recent systematic literature review of 52 papers on resilience in sport and work proposed “a state-like conceptualisation of resilience involving processes of adaptation and growth from stressors” (Bryan et al., 2019). The authors describe a shift in conceptualizing resilience as a trait toward a state-like process which is a dynamic construct. Van Breda (2018) also supports the need for an explanation of factors that mediate between adversity and outcomes, and suggests that “resilience is a process that leads to an outcome, and the central focus of resilience research is on the mediating processes” (p. 4).

Factors contributing to personal resilience include “self-confidence and high levels of self-efficacy” (Mahdiani & Ungar, 2021, p. 149). Bryan et al. (2019) summarized resources needed for building and maintaining resilience as “support; self-efficacy; optimism; coping skills; motivation; perspective; self-regulation; hardiness; proactiveness; adaptability; sense of control; and positive mind-set” (p. 81). Van Breda (2018) identified “grit, intelligence, problem-solving skills, emotional regulation, motivation to succeed, faith and hope” as individual factors contributing to resilience processes or protective resources (p. 7). These studies highlight multi-faceted nature of resilience.

Terms such as resilience and coping are frequently used in a similar way; however, conceptual distinction between the two needs to be clarified. Fletcher and Sarkar (2013) explain that “resilience *influences* how an event is appraised, whereas coping refers to the strategies employed *following* the appraisal of a stressful encounter” (p. 16; authors’ italics). Bryan et al. (2019) explain further: “coping is characterised as a responsive ability to reduce or tolerate external demands, where resilience is characterised prior to emotional and coping responses and is evident by its positive process of learning adaptions from all adverse experience” (p. 87). Positive adaptations do not always occur in an immediate response to a calamity. Research suggests that “personal growth requires a period of decline in functioning if new capacities are to be realized” (Mahdiani & Ungar, 2021, p. 148).

Pratt (2015) questioned the interpretation of resilience in the context of the cultural economy. He warned that the typical approach of “making do with less” leads to increased precarity of cultural work. Instead, “a form of local capacity building applied to the particularities of the cultural economy, and place, offer a more productive strategy for resilience” (p. 62). This might include more funding for experimentation and breadth together with welfare support to create growth in the cultural economy.

Little is known regarding resilience and coping strategies of professional musicians, despite much research into their generally poor physical and mental health (e.g., Ackermann et al., 2014; Kenny et al., 2014), music performance anxiety (e.g., Zhukov, 2019), and injury and pain during playing (e.g., Cruder et al., 2017). Health awareness and injury attitudes among tertiary students have been demonstrated to be poor (Rickert et al., 2015), with repeated calls made for greater education in healthy practice in professional training programs. A recent survey of 64 student and professional classical musicians showed that participants with higher resilience and better physical health were less likely to flag mental health issues; however, overall, high levels of depression and anxiety were reported, particularly among the music students (Kegelaers et al., 2021). Research demonstrates that the precarity of musicians’ careers has only been exacerbated through the global pandemic (Spiro et al., 2021). Notwithstanding this, in the face of severe limitations to established and well-practiced collaborative practices, some collaborative musicians have been found to demonstrate resilience through engaging with new modalities for making music and venturing into previously unexplored musical terrain (Fram et al., 2021). The circumstances of the pandemic might be viewed as “a period of decline” from which “new capacities” might be realized (Mahdiani & Ungar, 2021, p. 148). We therefore draw on the lens of recent resilience theory to interrogate the experiences of freelance creative collaborative musicians during lockdowns.

## Methodology

### Research site

A Melbourne-based contemporary music ensemble was the case for this research (Yin, 2018). The group consists of several core musicians who typically invite a guest artist to join them for a specific project where new music is generated over one day and is performed to a live audience at the end of the day. This particular group was chosen because previous research has not specifically focused on musicians who create new work. Arguably, the experience of freelance creative collaborative musicians requires considerable resilience to navigate the challenges of career self-management.

The usual approach to their ensemble work had been severely disrupted during Melbourne lockdowns, forcing the group to pivot to new ways of creating and collaborating. These included undertaking initial planning meetings via Zoom, and creating and recording individual contributions to the collaboration over the period of a single day while working in isolation, with contributions being subsequently edited into a final work. This article reports on two creative collaborative projects.

### Data collection

Ethical clearances were obtained from an Australian university prior to the commencement of research. A qualitative approach was chosen to obtain rich data and multiple points of view from the participants. Individual interviews provided an opportunity to probe deeply, ask for clarifications, and confirm understandings. Interviews were audio recorded and transcribed by a professional transcription company. Member-checking of interviews provided further opportunity for participants to reflect on their perspectives and the data provided.

Members of the group gave their informed consent to be interviewed twice during April to September 2021, regarding two different projects. Six participants were interviewed regarding the first project and six regarding the second, with three core members being the same (interviewed on both occasions) and three project contributors different between the two projects (making a total of 12 interviews with nine participants). Due to the bespoke nature of the ensemble, specific information regarding age, sex, and ethnicity has been withheld to protect the participants. The participants ranged from mid- to late-career, and some had caregiving responsibilities for children. Only two reported receiving temporary state-funded support through JobKeeper.

Semi-structured interviews probed individual music career histories as a creative collaborative musician, personal and professional Covid-related impact, lockdown adaptations in creative practice, and future outlook. This article focuses on the impacts of recurring lockdowns on the personal and professional lives of these freelance creative collaborative musicians.

### Thematic analysis

The interviews were initially coded by one researcher under three main themes: personal impacts of lockdowns, professional impacts, and future outlook (Braun & Clarke, 2012). The emerging categories and sub-categories were refined through iterative discussions between the research team (Maxwell & Miller, 2008). Participant quotes were used to demonstrate the trustworthiness of findings (Given & Saumure, 2008).

## Findings and discussion

### Theme 1: Professional impacts of recurring lockdowns

When discussing the professional impact of recurring lockdowns, the participants highlighted three major issues: loss of work, loss of artistic identity, and professional coping strategies that sustained them during pandemic.

#### Loss of work

The participants described the first few weeks of pandemic as endless emails canceling work, trips, and recording studio sessions, with their professional lives “crumbling down.” A nationally significant art gallery that supported 2,500 artists closed after 14 years of operation. As one respondent reported, “The first thing that happened is that a lot of my work just disappeared. My wife’s work disappeared completely, she’s a singer and all of her work is live performance.” Tours that took years of planning were canceled, leaving musicians in limbo: “Not being able to leave the country or the state has changed a huge amount of my life.”

Being a freelance musician during the pandemic was particularly “daunting” with the financial impact of no regular income. In the state of Victoria, there were blanket bans on face-to-face work; for example, “the Rising Festival was cancelled the day after opening night.” One participant described it as a “roller coaster time”:

I was in a quarantine and couldn’t leave my house for two weeks. And then we went immediately into rehearsals for the project, and then our project got shut down due to the next lockdown about 10 days later.

#### Loss of artistic identity

The sudden interruption of normal working routines resulted in uncertainty about the future and made musicians feel like they were “artists in theory and not in practice.” One participant reported that “it felt very artificial to be working on projects that had no obvious future . . . it suddenly became a little meaningless to be making work when I couldn’t be making it with other people.” Another musician had a complete reassessment of their career: “It definitely made me re-evaluate everything, my future, my practice, where the opportunities are going to lie and perhaps where they won’t lie.”

The respondents described the cumulative effects of lockdowns on the arts community as “apathy” and “creative doldrums.” One person reflected, “When I looked back and particularly in the second lockdown, I realized what a dark place I’ve gone to. I had stopped doing a lot of the things that I usually love.”

The participants found the loss of professional connections “very disorientating.” One musician explained, “Going and seeing work and attending festivals and having that social network, not online but physically, is a really important part of how you orient yourself in your career. That had just disappeared, basically.”

#### Professional coping strategies

To sustain themselves during recurring lockdowns, the participants adopted a range of professional coping strategies. Focusing on “small wins” while working from home on new projects was one approach, as reported by a participant:

If I could record five minutes of music, or edit five minutes of music, answer some emails, and have a phone call, it was still a goal and it was important to be satisfied with the fact that I could do something.

Maintaining instrumental practice and mastering other instruments was seen as an opportunity to stay fit and extend current performance skills:

I have a 30-minute to an hour-long routine that I do if I don’t have anything else currently to practise. I think I wanted to make sure that I didn’t come out the other side not able to play because of the lockdown.

Developing new ideas for future projects helped the participants remain creative. For example, one person used the 1-hr of permitted exercise to brainstorm ideas for future projects with others: “I’ve been meeting up with a range of different artists, walking down the river or walking through the neighbourhood and sometimes just catching up, other times planning projects, and just digesting ideas.” Others viewed the lockdowns as an opportunity to develop their own work: “It’s given me time to be able to focus on some bigger ideas with greater generosity.”

Overall, the findings demonstrate a pattern of loss followed by some recovery characterized by specific coping strategies. The findings showed that professional impacts experienced by Australian freelance creative collaborative musicians were similar to other musicians around the world, with cancelations of performances and tours resulting in loss of work and income (Rusak et al., 2021; Spiro et al., 2021). The loss of artistic identity reported here was previously highlighted in a study of orchestral musicians in the United Kingdom (Cohen & Ginsborg, 2021). One of the professional coping strategies identified by the participants was maintaining their playing skills through regular practice and learning to play new instruments. Similarly, Cohen and Ginsborg (2021, 2022) reported motivation to play, practice, and expand musical skills as sub-themes in their analysis of interviews of professional orchestral musicians. Fram et al. (2021) also reported that some musicians explored new ways of being collaborative and expanded their practices during the pandemic, following an initial period of acute loss. Other professional coping strategies included working on new projects from home and developing ideas for future projects. Maximizing current professional opportunities and generating new opportunities were previously reported in research (Cohen & Ginsborg, 2021; Spiro et al., 2021).

The perspectives of creative collaborative musicians regarding the impacts of the Covid-19 lockdowns on their professional lives reported here demonstrate their *resilience* in spite of difficult circumstances. Mahdiani and Ungar (2021) suggest that personal growth may emerge from adversity, when resilience resources are available.

Initially, cancellations of performances had resulted in a loss of artistic identity for the musicians in this study. Their appraisal of the drastic pandemic situation over time led to professional coping strategies as well as long-term positive adaptations in the form of exploration and expansive practices. As Mahdiani and Ungar (2021) explain, “exposure to significant threat or severe adversity and the achievement of positive adaptation are consistent attributes of resilience” (p. 148). Similarly, Cohen and Ginsborg (2022) reported post-traumatic growth in mid-career musicians who were “open to new possibilities” and “discovering personal strengths” post-pandemic (pp. 10–11). In this vein, professional coping strategies went beyond maintaining skills and collaborative networks, extending to developing new playing skills, generating ideas for new projects, and maximizing opportunities.

### Theme 2: Personal impacts of recurring lockdowns

Participant reflections on personal impacts of recurring lockdowns focused on three areas: lockdown stressors, personal coping strategies, and relationships.

#### Lockdown stressors

Lockdown stressors were front-and-center when the participants reflected on the personal impacts of the pandemic. Having observed friends and colleagues battling Covid-19, the participants were afraid of the disease and recognized its severity: “It made me quite fearful of getting it. And it made me quite angry at people who weren’t taking lockdowns and safety measures seriously. It made me very wary about big groups.”

Respondents commented on feeling worn out and flat:

The thing that is missing in my life is adrenaline. And that used to be a huge part of the chemical makeup of a performer, the extreme pressure of building up towards a performance that includes late nights, physical exertion, nerves, adrenaline, and then the huge sense of achievement. When those things are missing, it’s really hard, it’s flatlined and somewhat boring.

The lockdowns affected some musicians with self-doubts, as one reflection shows: “It’s definitely affecting people mentally, and it’s limiting their belief in themselves and their ability to share their music.” Others recognized the diversity of responses: “Everybody is living in completely different environments, not everyone has got the same coping strategies. We’re in the same storm, but everyone’s got completely different boats that they’re riding the storm in.”

#### Personal coping strategies

Some participants’ personal coping strategies “centred around food and cooking.” Exercise and hobbies served the purpose of filling in time during lockdowns and also giving a sense of achievement and control that was missing from everyone’s lives at the time. For example, one participant reported, “In the first lockdown this time last year, we were doing lots of hour-walks or two-hour walks with the family around the neighbourhood looking for flowers, wild or otherwise, cultivated or grown.” Another participant preferred indoor activities, “I did a lot of jigsaws, colouring in, played a lot of cards and I’ve found time to read for myself again.”

Helping others in more difficult circumstances gave meaning and purpose for one participant: “I was doing some of that emotional labour heavy-lifting and checking in with friends and family and making sure that people are OK because I know I was not struggling as much as other people were.”

Planned recreational activities served as “markers in time.” One participant described regular themed nights:

On Monday nights, we used to have pasta and listen to this podcast. Tuesday night my partner used to play Dungeons and Dragons on the internet with his brother and their friends. Wednesday night used to be wine and cheese night. Friday night would always be delivery and fizzy wine night. Sunday nights became black-and-white movie nights.

#### Relationships

Maintaining relationships with friends and family online or in person played an important role in supporting the participants emotionally during lockdowns, as seen in the quotations below:

I felt that a conversation with a friend, a bit of a heart-to-heart, just hearing how they were going and how are you coping and what are you doing was also an important, a legitimate part of the day and that it was OK to see that as a successful day if you’d had a conversation like that, that it wasn’t just wasting time.

I was happy to not go and see my dad and give him a fatal disease, I could only talk to him on Zoom. I was missing seeing him but it was so obviously the better thing for him.

The findings show that the main personal impacts raised by the participants were fear of illness, exhaustion, and doubts regarding careers. Freelance cultural workers in the United Kingdom similarly reported “increased psychological and physical symptoms as a result of their changing working lives and the consequences of these changes on their professional identities” (Warran et al., 2022, p. 16). Rusak et al. (2021) reported that “the disruption to performances during lockdown led performers to re-evaluate their artistic practice, whether through having a break or reassessing their career paths” (p. 1).

The participants discussed personal coping strategies of filling the time at home during lockdowns with purposeful activities such as cooking, exercise, hobbies, helping others, and planned recreation. This finding aligns with previous research where a synthesis of 16 studies demonstrated that “exercise, yoga, progressive muscle relaxation, and listening to relaxing music” had beneficial effects in promoting well-being during lockdowns (Puyat et al., 2020, p. 2). Cohen and Ginsborg (2022) also highlighted routines and physical exercise as coping strategies used by orchestral musicians to maintain well-being during the pandemic.

Focus on relationships through online social interactions with family and friends was also important for the participants. Similarly, Cohen and Ginsborg (2022) reported that orchestral musicians “valued support networks of friends, family, and colleagues” during the pandemic (p. 8). Warran et al. (2022) also described a greater engagement with family and friends in the changing attitudes to work of freelance cultural workers in the United Kingdom.

The participants described processes of adaptation to lockdown stressors (fear of infection, frustration, boredom, exhaustion, and doubts regarding careers) and demonstrated resilience on a personal level. These included being proactive through purposeful activities for self-regulation and sense of control, helping others and maintaining close relationships for emotional support. Research highlighted such resources as factors in resilience building and maintenance (Bryan et al., 2019).

### Theme 3: Future outlook

The participants’ future outlook focused on three issues: developing new professional skills and future directions, positive future outlook, and negative future outlook.

#### Developing new professional skills and future directions

The participants stated that the pandemic forced them to learn new technological skills such as “communicating online or having meetings online or teaching online.” New music-making opportunities necessitated development of new skills: “Livestreaming mix sessions was an innovation that was crucial to collaboration in that time. It made me hone in a little bit more on technical aspects of my studio.”

When planning for future projects, respondents tried to think laterally and “do something that hasn’t existed before.” As one participant reflected, “I’m putting a lot more of my energy into the planning stages at the moment, into fostering those relationships, and into looking at how we maybe find some projects and into dreaming up what might be next.”

In the absence of performance opportunities, greater effort went into administration and grant-writing:

My focus this year has shifted from performance to more of admin and more about how we emerge from this time ready to do great projects and to talk about our work. I was very lucky earlier this year to get some funding towards mentoring, so I’ve been spending a lot of time with four different mentors lately.

Maintaining engagement within the community and creating new opportunities was viewed as essential and productive: “Being in one place for a significant amount of time, not leaving, re-engaging with community, especially with a community undergoing significant hardship has led to new friendships, and new routines, and new collaborative outcomes with various different people.” For example, one participant reported:

I developed an online program that we ran for high school students in the September school holiday. So specifically for students who were still in the lockdown and who basically spent a week with us getting creative prompts from a range of different performers and composers.

#### Positive future outlook

The participants with a positive future outlook discussed the need for industry innovation, “to dig deeper, become more engaging, more relevant to contemporary society, more viable in a variety of ways.” For example, they highlighted the potential for greater support for the small-to-medium arts sector versus the bulk of the funding being channeled into large national companies:

The pandemic is going to take a little bit of the appetite out of just subsidizing opera and symphony orchestras to high degrees outside of the pandemic, which I think is a great opportunity for a diversified sector to come through. I do think more support and more opportunity for the small-to-medium sector would create a much more interesting musical landscape in Australia.

The respondents commented on the public’s appetite for the return to live performances: “I can’t wait to be able to go to a live gig, to go to the theatre, even to wander around an art gallery and have some toddler crash into me as they are screaming and yelling.” Face-to-face live interactions were seen as vital for creative work:

You need to be in a room and you need to feel that response from that other person, you need to be in those free-form conversations where you’re just like, all cylinders firing. And I think artists can’t wait to get back in there.

Screen fatigue was cited as a factor: “Everyone’s pretty sick of being on their computer and being on Zoom.” As one participant explained, “People went digital because they had to, but I don’t think they will end up being a substitute for the live performance.”

#### Negative future outlook

Some older participants exhibited a negative future outlook, displaying symptoms of the “great resignation”: “I’m just really demoralized, and I’m just really, really tired. I’m re-evaluating my career choices right now, I’m exhausted and I kind of feel like I just need out.” Others were concerned about the loss of cultural life in the future:

It’s difficult to see concrete ways forward as an independent artist, but the real sadness is seeing the sector lose talent now. There are a lot of people who are choosing to leave the sector. I feel a little more cautious, and maybe a little sadder about the state of the industry and where some of my colleagues are at.

Planning future projects and developing new technological and administrative skills to support these new ideas led to a positive future professional outlook displayed by many of the participants in this study. Similar findings have been reported by Spiro et al. (2021) who identified a theme of professional and personal opportunities that included developing new possibilities and new skills. Cohen and Ginsborg (2022) also described musicians “acquiring new online music production skills” during the pandemic (p. 10). Gee and Yeow (2021) suggested that to sustain music careers a wide range of “soft” organizational skills are essential in addition to musical expertise and technical skills.

North American research flagged the need for innovation in the industry, concluding that to survive post-Covid recession “symphony orchestras will need to practice cost-cutting measures, while reinventing themselves through innovative programmes, including increased streaming platforms” (Pompe & Tamburri, 2023, p. 1). Similarly, UK research highlighted the importance of “widening audiences and participation to strengthen the cultural industries” and “maintaining adaptive and hybrid creative models” (Shaughnessy et al., 2022, pp. 5–6).

In contrast, Cohen and Ginsborg (2022) reported that late-career musicians aged more than 66 feared that “they might already, *de facto*, have retired” (p. 12). Similarly, Flore et al. (2023) described creative workers’ “grief and loss for their, and the industry’s, futures” (p. 208). It was not surprising that some older participants in our study displayed a negative future outlook and symptoms of the Great Resignation (Sull et al., 2022).

The participants demonstrated many interpersonal resources that contributed to building and maintaining resilience: personal growth in acquiring new technological skills and innovative approaches to future projects, problem-solving skills to generate new ideas for future projects, and coping strategies to regulate emotions and sustain optimism and hope. These characteristics have been highlighted by research as necessary elements of resilience (Bryan et al., 2019; Van Breda, 2018).

## Conclusions and implications

The resilience demonstrated by the creative collaborative musicians in this study stands in contrast to the lack of support provided by the Australian Federal government to small arts organizations during the pandemic. This suggests that while resilience may be a positive skill for musicians, in this instance, it is something that is enforced by the economic and cultural conditions in the sector (Pratt, 2015). Policy recommendations for public-led reconstruction include funding for community arts and artists, public streaming platforms, arts education, and improved pay and conditions of the arts sector workers (Pennington & Eltham, 2021). Similarly, in the United Kingdom, Comunian and England (2020) argue that the fact that the arts sector shows resilience during difficult times leads to under-investment in the sector. It is urgent that “policymakers should rethink the rhetoric of resilience in [creative and cultural work] and consider the longer-term implications of transformations in employment structures during and following periods of economic crisis” (p. 122). Our study contributes to the international debate on resilience in the cultural sector and recommends that future policy transformations provide security and stability for creative artists and enable them to flourish.

This study documents resilience among a group of creative collaborative musicians during recurring lockdowns, and the findings are necessarily limited to the specific case. In spite of lockdown stressors (fear of infection, frustration, boredom, exhaustion, and doubts regarding careers), the ensemble managed to create and disseminate new work. The capacity to bounce back from loss and to even expand their collaborative practices was perhaps only possible owing to a number of resilience resources, including a well-established collaborative network, strong interpersonal relationships, creative skills, and coping strategies. It is also important to note that positive adaptation, labeled as “resilience,” may in fact be masking vulnerability and that the far-reaching effects of the Covid-19 pandemic remain, at the time of writing, largely unknown. As Mahdiani and Ungar (2021) state, “those who evidence successful adaptation often struggle with covert psychological difficulties over time, such as problems of depression and posttraumatic stress” (p. 148).

The findings represent perspectives of members of a specific group of music creatives who have worked together for a number of years. The ensemble’s modus operandi of creating new work with new collaborators over one day and presenting live to an audience on the same evening has prepared the participants to work under pressure, be flexible and open to new ideas, deal with the unexpected, and trust each other. Furthermore, a sustained history of working in the small-medium arts sector, where generating one’s own creative projects is the prime focus, may well have contributed to this group’s more positive reports.

The strong professional and personal relationships have allowed the ensemble to pivot in new directions and withstand the drastic impacts of lockdowns such as social isolation and restriction on movement. As research has pointed out, a sustainable career in music requires

awareness of self and musical identitie(s), ability to (practically, emotionally, financially) handle concurrent multiple job roles, hard and soft skill sets which can adapt to a range of environments and demands, as well as a clear baseline of expert knowledge which can be adapted to specific contexts. (Gee & Yeow, 2021, p. 351)

Previous research has focused on freelancing musicians working in larger organizations such as orchestras (e.g., Cohen & Ginsborg, 2021, 2022). This study provides new evidence for the cultural policy and arts managers regarding the professional and personal impacts of lockdowns on freelance creative collaborative musicians and highlights the urgent need for state support of the small-medium arts sector.

For us as higher education music educators and researchers, concerns about the impacts of lockdowns on current undergraduate music students and recent graduates starting their careers remain the focus for the profession if we are to sustain the artistic practice and cultural lives of contemporary society. As Canham (2023) points out, the pandemic has forced “a paradigm shift in music careers education and research: things to think about, things to leave behind, and things to do differently” (p. 3). We urge higher education providers to prioritize digital technology and recording training and place a greater emphasis on physical and psychological well-being of students.

## References

[bibr1-1321103X231186952] Ackermann B. J. Kenny D. T. O’Brien I. Driscoll T. R. (2014). Sound practice-improving occupational health and safety for professional orchestral musicians in Australia. Frontiers in Psychology, 5, Article 973. 10.3389/fpsyg.2014.00973 25249990 PMC4158789

[bibr2-1321103X231186952] Barrett M. S. Creech A. Zhukov K. (2021). Creative collaboration and collaborative creativity: A systematic literature review. Frontiers in Psychology: Systematic Review, 12, 713445. 10.3389/fpsyg.2021.713445 PMC8380918 34434151

[bibr3-1321103X231186952] Braun V. Clarke V. (2012). Thematic analysis. In Cooper H. Camic P. M. Long D. L. Panter A. T. Rindskopf D. Sher K. J. (Eds.), APA handbook of research methods in psychology: Research designs: Quantitative, qualitative, neuropsychological, and biological (Vol. 2, pp. 57–71). American Psychological Association. 10.1037/13620-000

[bibr4-1321103X231186952] Bryan C. O’Shea D. MacIntyre T. (2019). Stressing the relevance of resilience: A systematic review of resilience across the domains of sport and work. International Review of Sport and Exercise Psychology, 12(1), 70–111. 10.1080/1750984X.2017.1381140

[bibr5-1321103X231186952] Canham N. (2023). Living with liminality: Reconceptualising music careers education and research. Research Studies in Music Education, 45(1), 3–19. 10.1177/1321103X221144583

[bibr6-1321103X231186952] Cohen S. Ginsborg J. (2021). The experiences of mid-career and seasoned orchestral musicians in the UK during the first COVID-19 lockdown. Frontiers in Psychology, 12, Article 645967. 10.3389/fpsyg.2021.645967 33897549 PMC8062715

[bibr7-1321103X231186952] Cohen S. Ginsborg J. (2022). One year on: The impact of COVID-19 on the lives of freelance orchestral musicians in the United Kingdom. Frontiers in Psychology, 13, Article 885606. 10.3389/fpsyg.2022.885606 35712210 PMC9196900

[bibr8-1321103X231186952] Comunian R. England L. (2020). Creative and cultural work without filters: Covid-19 and exposed precarity in the creative economy. Cultural Trends, 29(2), 112–128. 10.1080/09548963.2020.1770577

[bibr9-1321103X231186952] Cruder C. Falla D. Mangili F. Azzimonti L. Araújo L. Williamon A. Barbero M. (2017). Profiling the location and extent of musicians’ pain using digital pain drawings. Pain Practice, 18, 53–66. 10.1111/papr.12581 28466572 PMC6849566

[bibr10-1321103X231186952] Fletcher D. Sarkar M. (2013). Psychological resilience: A review and critique of definitions, concepts, and theory. European Psychologist, 18(1), 12–23. 10.1027/1016-9040/a000124

[bibr11-1321103X231186952] Flore J. Hendry N. A. Gaylor A. (2023). Creative arts workers during the Covid-19 pandemic: Social imaginaries in lockdown. Journal of Sociology, 59(1), 197–214. 10.1177/14407833211036757 36873054 PMC9892877

[bibr12-1321103X231186952] Fram N. R. Goudarzi V. Terasawa H. Berger J. (2021). Collaborating in isolation: Assessing the effects of the Covid-19 pandemic on patterns of collaborative behavior among working musicians. Frontiers in Psychology, 12, Article 674246. 10.3389/fpsyg.2021.674246 34349700 PMC8326970

[bibr13-1321103X231186952] Gee K. Yeow P. (2021). A hard day’s night: Building sustainable careers for musicians. Cultural Trends, 30(4), 338–354. 10.1080/09548963.2021.1941776

[bibr14-1321103X231186952] Given L. M. Saumure K. (2008). Trustworthiness. In Given L. M. (Ed.), The Sage encyclopedia of qualitative research methods (pp. 896–987). Sage.

[bibr15-1321103X231186952] Kegelaers J. Schuijer M. Oudejans R. R. D. (2021). Resilience and mental health issues in classical musicians: A preliminary study. Psychology of Music, 49(5), 1273–1284. 10.1177/0305735620927789

[bibr16-1321103X231186952] Kenny D. T. Driscoll T. Ackermann B. (2014). Psychological well-being in professional orchestral musicians in Australia: A descriptive population study. Psychology of Music, 42(2), 210–232. 10.1177/0305735612463950

[bibr17-1321103X231186952] Mahdiani H. Ungar M. (2021). The dark side of resilience. Adversity and Resilience Science, 2, 147–155. 10.1007/s42844-021-00031-z

[bibr18-1321103X231186952] Maxwell J. A. Miller B. A. (2008). Categorizing and connecting strategies in qualitative data analysis. In Hesse-Biber S. N. Leavy P. (Eds.), Handbook of emerging methods (pp. 461–477). Guilford Press.

[bibr19-1321103X231186952] Moylan K. (2023). “Welcome to a Coronavirus production”: Beyond bows and arrows’ Indigenous on-air community-building during lockdown. International Journal of Cultural Studies, 26(1), 52–68. 10.1177/13678779221123079

[bibr20-1321103X231186952] Pennington A. Eltham B. (2021). Creativity in crisis: Rebooting Australia’s arts and entertainment sector after Covid. The Centre for Future Work at the Australia Institute.

[bibr21-1321103X231186952] Pompe J. Tamburri L. (2023). The symphony orchestra in the time of Covid-19: Will American orchestra rise from the ashes? Cultural Trends, 32(1), 35–51. 10.1080/09548963.2022.2044266

[bibr22-1321103X231186952] Pratt A. C. (2015). Resilience, locality and the cultural economy. City, Culture and Society, 6(3), 61–67. 10.1016/j.ccs.2014.11.001

[bibr23-1321103X231186952] Puyat J. H. Ahmad H. Avina-Galindo A. M. Kazanjian A. Gupta A. Ellis U. Ashe M. C. Vila-Rodriguez F. Halli P. Salmon A. Vigo D. Almeida A. De Bono C. E. (2020). A rapid review of home-based activities that can promote mental wellness during the COVID-19 pandemic. PLOS ONE, 15(12), Article e0243125. 10.1371/journal.pone.0243125 PMC7714353 33270755

[bibr24-1321103X231186952] Rickert D. L. L. Barrett M. S. Ackerman B. J. (2015). Are music students fit to play? A case study of health awareness and injury attitudes amongst tertiary student cellists. International Journal of Music Education, 33(4), 426–441. 10.1177/0255761415582343

[bibr25-1321103X231186952] Rusak H. Goh T. Barbe F. Newman R. Blevins P. (2021). Breathing through the pandemic: Performing arts challenges and responses to the mental health implications of Covid-19. Edith Cowen University. https://www.waapa.ecu.edu.au/__data/assets/pdf_file/0006/960387/Breathing-through-the-pandemic.pdf

[bibr26-1321103X231186952] Shaughnessy C. Perkins R. Spiro N. Waddell G. Campbell A. Williamon A. (2022). The future of the cultural workforce: Perspectives from early career arts professionals on the challenges and future of the cultural industries in the context of COVID-19. Social Sciences & Humanities Open, 6(1), Article 100296. 10.1016/j.ssaho.2022.100296 35602244 PMC9113958

[bibr27-1321103X231186952] Spiro N. Perkins R. Kaye S. Tymoszuk U. Mason-Bertrand A. Cossette I. Glasser S. Williamon A. (2021). The effects of COVID-19 lockdown 1.0 on working patterns, income, and wellbeing among performing arts professionals in the United Kingdom (April–June 2020). Frontiers in Psychology, 11, Article 594086. 10.3389/fpsyg.2020.594086 33643111 PMC7902701

[bibr28-1321103X231186952] Sull D. Sull C. Zweig B. (2022). Toxic culture is driving the Great Resignation. MIT Sloan Management Review, 63(2), 1–9.

[bibr29-1321103X231186952] Van Breda A. D. (2018). A critical review of resilience theory and its relevance for social work. Social Work, 54(1), 1–18. 10.15270/54-1-611

[bibr30-1321103X231186952] Vincent C. (2023). The impacts of digital initiatives on musicians during Covid-19: Examining the Melbourne Digital Concert Hall. Cultural Trends, 32(3), 247–263. 10.1080/09548963.2022.2081488

[bibr31-1321103X231186952] Warran K. May T. Fancourt D. Burton A. (2022). Understanding changes to perceived socioeconomic and psychosocial adversities during COVID-19 for UK freelance cultural workers. Cultural Trends. Advance online publication. 10.1080/09548963.2022.2082270 PMC11005917 38601794

[bibr32-1321103X231186952] Yin R. K. (2018). Case study research and applications: Designs and methods (6th ed.). Sage.

[bibr33-1321103X231186952] Zhukov K. (2019). Current approaches for management of music performance anxiety: An introductory overview. Medical Problems of Performing Artists, 34(1), 53–60. 10.21091/mppa.2019.1008 30826822

